# Leftover Food as a Sustainable Source of Astaxanthin Through Fermentation Using *Phaffia rhodozyma*

**DOI:** 10.3390/foods14071232

**Published:** 2025-03-31

**Authors:** Rossella Vadalà, Eleonora Di Salvo, Laura De Maria, Giovanna Lo Vecchio, Giovanni Bartolomeo, Rita De Pasquale, Claudia Genovese, Nicola Cicero, Rosaria Costa

**Affiliations:** 1 Department of Biomedical Dental Morphological and Functional Imaging Sciences, University of Messina, 98100 Messina, Italy; rvadala@unime.it (R.V.); eleonora.disalvo@unime.it (E.D.S.); laura.demaria@studenti.unime.it (L.D.M.); giovanna.lovecchio@unime.it (G.L.V.); gbartolomeo@unime.it (G.B.); rita.depasquale@studenti.unime.it (R.D.P.); costar@unime.it (R.C.); 2 National Research Council, Institute for Agricultural and Forest Systems in the Mediterranean, 95128 Catania, Italy; claudia.genovese@cnr.it; 3 Science4life S.r.l. Start Up, 98168 Messina, Italy

**Keywords:** food waste, astaxanthin, fermentation, *Phaffia rhodozyma*, bioreactor, yield, sustainable approach

## Abstract

Natural astaxanthin is a bioactive with high antioxidant power, widely suitable for many applications. This study explores the potential of leftover food as a sustainable and low-cost substrate for producing astaxanthin via direct fermentation using *Phaffia rhodozyma*. The pretreated and characterized raw materials were fermented in a lab-scale bioreactor under optimized process conditions. The entire process (168 h) was monitored in terms of reducing sugar consumption, yield, and productivity of astaxanthin. The implemented experimental plan achieved high astaxanthin yield and producticity, namely, 230 mg·L^−1^ and ~1.6 mg·L^−1^·h, which were attained at 150 h, respectively, with a substrate consumption of around 90% for all samples. The natural astaxanthin obtained showed interesting antioxidant activity, exhibiting a radical scavenging activity of more than 65%, which was evaluated with a DPPH assay. This process not only offers a promising solution for leftover food valorization but also provides a sustainable approach to producing bioactive compounds with significant health value, paving the way for further industrial applications in food, pharmaceutical, and cosmetic sectors.

## 1. Introduction

Astaxanthin (3,3′-dihydroxy-β,β-carotene-4,4′-dione) is a pigment belonging to the xanthophyll family, oxygenated derivatives of carotenoids. Much evidence demonstrates the high antioxidant activity of this molecule, which has been found to be up to 10 times greater than that of other carotenoids and even 100 times higher than that of α-tocopherol [1]. The significant antioxidant action of astaxanthin, which structurally consists of a chain of polyenes with two terminal benzene rings, derives from its ability to remove free radicals from a system, reacting with them to produce harmless compounds or to block oxidation reactions. Such interactions induce beneficial effects on human health, particularly in combating cardiovascular disease, retinopathies, tumors, and immune system diseases [2]. Recent studies also confirm that the antioxidant action of astaxanthin inhibits the activity of *Helicobacter pylori*, protecting the gastrointestinal tract from the onset of gastritis, peptic ulcers, and stomach carcinomas [3]. For these reasons, the demand for this substance from various sectors, such as fish nutrition, cosmetics, pharmaceuticals, and food, is continually growing [4]. According to Grand View Research, the global astaxanthin market recorded a value of USD 1.0 billion in 2019, with an expected growth of 16.2% by 2027. Methods for the production of astaxanthin can be divided into two main categories: chemical synthesis and extraction, which can occur from aquatic animals, or microbiological means using basidiomycetes such as *Phaffia rhodozyma* or marine algae such as *Haematococcus pluvialis* [5,6,7,8]. Currently, 95% of the astaxanthin on the market is synthetic. However, synthetic production has significant limitations, such as poor environmental sustainability of production processes and low purity due to the fact that synthetic molecules present a combination of different isomerisms and an antioxidant capacity that is approximately 20 times lower than that of natural astaxanthin [6]. The most significant criticism is that the use of synthetic molecules is only permitted in animal nutrition, through inclusion in the diet of salmon, crustaceans, and farmed fish, with the dual purpose of promoting a red-orange pigmentation in these species highly appreciated by consumers and ensuring a functional and nutritional component for good growth and reproduction [9]. The pharmaceutical, cosmetic, and food sectors can exclusively use naturally derived astaxanthin. For these reasons, the search for natural sources of astaxanthin and extractive processes transferable on an industrial scale is currently of great interest to the scientific community. Weaknesses dominating this area of development are still represented by the limited natural sources and the cost of raw materials, as well as the poor economic sustainability and final yield of production processes. The chemical synthesis of 1 kg of astaxanthin has an approximate cost of USD 1000, while microbiological extraction can range from a minimum of USD 2500 to a maximum of USD 7000 per kg of substance [6]. It has been widely documented that *P. rhodozyma* is the most effective microorganism in astaxanthin production, presenting undeniable advantages such as a fast growth rate, simple culture conditions that do not require the use of lighting, and high proliferation density [10]. Astaxanthin is the main pigment of *Phaffia*, which on average contains 500 µg/g of total carotenoids, of which 40–95% consists of astaxanthin. This strain is characterized by the ability to produce surface cells associated with amyloid compounds and the ability to ferment sugars and assimilate carbon compounds such as D-glucose, maltose, sucrose, cellobiose, and hydrolyzed urea [11]. Despite the high scientific interest in this approach, technology transfer appears to be significantly delayed by the high costs of the processes. This issue can be partially overcome by using low-cost raw materials [12]. In this study, leftover food (LF) rich in lipids, proteins, and minerals was used as a medium for fermenting astaxanthin from *P. rhodozyma* in a bioreactor. LF is responsible for nearly two-thirds of total food waste generated around the globe, with levels of per capita food waste across countries and income groups being almost the same [13]. According to Andrews et al., leftovers are considered one of the core components of food waste, which includes the surplus of food produced during a meal, although LF valorization aimed at bioactive achievement is considered an under-researched area [14]. The overall objective of this work is to improve the economic and environmental impact of the microbiological extraction process of astaxanthin by achieving a good yield of this natural bioactive through a circular and low-cost process in which raw fermentation materials are recovered from leftover food, the latter being massive and difficult-to-manage waste [15]. The purpose of the work will be achieved through the implementation of an experimental design which is based on four crucial steps: (i) pretreatment and characterization of low cost raw materials (LF); (ii) optimization of strain culture conditions and direct fermentation variables; (iii) determination of content and productivity of astaxanthin obtained through the direct fermentation process; (iv) evaluation of the antioxidant activity of the produced astaxanthin.

## 2. Materials and Methods

### 2.1. Chemicals and Reagents

Solvents, including ethanol, n-hexane, n-heptane, n-hexadecane, chloroform, methanol, acetone (reagent grade), water, dichloromethane, acetonitrile, and methanol (HPLC grade), were purchased from J Merck Life Science (Merck KGaA, Darmstadt, Germany). Glycerol, sodium hydroxide (NaOH), and a Kjeldahl catalyst were supplied by Carlo Erba (Milan, Italy). Hydrogen peroxide (30% *v*/*v*), nitric acid (65% *v*/*v*) (trace metal analysis grade), and ultrapure water (resistivity of 10 mΩ·cm) were from Mallinckrodt Baker (Milan, Italy). Helium of 99.9995% purity was supplied by Rivoira gases (Milan, Italy). The following standards were provided by J.T. Baker Fisher (Milan, Italy): sorbitol, glucose, sucrose, mannose, rhamnose, arabinose, galacturonic acid, xylose, galactose, and fructose. Fatty acids methyl esters (FAMEs) reference standards (C4–C24) were purchased from Supelco (Bellefonte, PA, USA). Peptone, yeast extract, malt extract, and dextrose were purchased from Oxoid-Unipath LTD (Basingstoke, Hampshire, UK). The carrot extract (EXTZ-199) was purchased from Creative Enzymes (Shirley, NY, USA). Standard synthetic astaxanthin and standard HPLC- grade astaxanthin were also purchased from J Merck Life Science (Merck KGaA, Darmstadt, Germany).

### 2.2. Raw Material and Its Pretreatment

Food waste, consisting of leftover food (LF), was collected from the canteen of the University of Messina (Italy). The LF had a heterogeneous composition, which mainly consisted of rice, maize, and preboiled pasta, exhausted fats and oils, raw and cooked vegetables such as lettuce leaves, potatoes and spinach, beets, peas and carrots, and fruit peels of bananas, apples, and pears. For the study, two collections of LF were used on different days, resulting in two samples labeled LF1 and LF2, each one weighing 1 kg. The composition of the samples showed few quantitative and qualitative differences. Details regarding the composition of the collected samples are provided in Table S1 in Supplementary Materials. LF was pretreated according to the following steps: drying firstly in a conventional heater at 90 °C for 3 h and 60 °C for 7 h; finally, grinding to powder via a knife mill (Retsch Grindomix gm 200). The grain size was calculated by means of an optical granulometer (Haver Computerized Particle Analysis CPA 2-1, Haver & Boecker OHG, Oelde, Germany) and estimated as 1.4 mm. Subsequently, the powder was sonicated in an Ultrasonic Bath LBS1. The treatment was conducted under the following conditions: 20 kHz, 0.4 W/mL for 15 min, resulting in an increase in the demand for dissolved chemical oxygen of approximately 60%. After mixing, the LF powder was stored at −20 °C. Before the fermentation tests, the pretreated raw materials were dried at 90 °C for 1 h.

### 2.3. Characterization of Raw Material

Once pretreated, the LF was analyzed for the assessment of its physical-chemical properties, proximate composition (pH, moisture content and dry matters; ash, reducing sugar, lipid, protein and dietary fiber), and fatty acids quantification. From each LF1 and LF2 sample, five aliquots were taken and analyzed; each variable was determined in triplicate. The determination of the proximate composition was carried out following the AOAC official protocol of analysis [16].

#### 2.3.1. pH and Moisture

The pH was evaluated by using a digital portable pH meter (model 3510, Jenway (Cole-Parmer Ltd.), Staffordshire, United Kingdom). Moisture was determined according to AOAC Method 925.09 by oven drying the sample at 110 °C for 4 h and subsequently registering the sample weight loss [16].

#### 2.3.2. Dietary Fiber, Ash, and Protein

Dietary fiber (DF) was determined using AOAC Official Methods 985.29 and 991.43 and AACC Method 32-07.01, employing a Megazyme assay kit (International Ireland Ltd., Wicklow, Ireland) [16]. Two sample aliquots (1.0 g each) were simultaneously treated with α-amylase at 80 °C, followed by digestion with protease and amyloglucosidase at 60 °C. The resulting solutions were cooled to approximately 40 °C and treated with ethanol to precipitate the fiber. The residues were then filtered, washed with organic solvents, and dried, and their mean weight was calculated. One residue was incinerated at 500 °C until reaching a constant weight (~12 h) to determine ash content, while the other was analyzed for crude protein using AOAC Official Method 976.05 [16]. This involved digestion with sulfuric acid, copper (II) selenite dihydrate, and potassium sulfate using the SpeedDigester K-439 (Büchi, Switzerland), followed by analysis using the KjelMaster System K-375 equipped with a gas and vapor scrubber (Büchi, Switzerland). The resulting solution was treated with sodium hydroxide to release ammonia, which was titrated with hydrogen chloride to determine nitrogen content. The protein content (%) was calculated by multiplying nitrogen (%) by a conversion factor of 6.5. Therefore, dietary fiber was calculated as the mean weight of the dried residue minus the weight of protein and ash.

#### 2.3.3. Total Lipids and FAMEs

Lipid content was determined using AOAC Method 920.39. According to Costa et al., a sample aliquot (4 g) was extracted using the Folch method based on a 2:1 solution of CHCl_3_/MeOH. The mixture was then placed in an ice bath and stirred for 30 min. Successively, the suspension was transferred into a separating funnel and agitated for a couple of minutes. The upper layer was then centrifuged for 15 min at 3000 rpm. Finally, the bottom layer was evaporated to dryness and the upper layer subjected again to the extraction process for another two times [17]. Total lipids were quantified gravimetrically by using a pre-weighed balloon in the rotary evaporator, where the chloroform fractions were dried.

Prior to fatty acid determination, each lipid extract was subjected to transesterification by dissolution in n-hexane and the addition of a 2 N solution of potassium hydroxide in methanol. The hexane upper layer obtained after shaking was injected into a gas chromatograph (GC) equipped with a split/splitless injector and linked to a flame ionization detector (FID) (Dani Master GC1000, Dani Instrument, Milan, Italy). The instrument was equipped with a Supelco SLB-IL100 capillary column (60 m × 0.25 mm ID, 0.20 μm film thickness, Supelco, Sigma Aldrich, Bellefonte, Pennsylvania, USA), with an oven temperature program ranging from 130 to 210 °C (10 min holding) at a rate of 2 °C/min. Injector and detector temperatures were maintained at 220 and 240 °C, respectively. Helium (He) served as the carrier gas with a linear velocity of 30 cm/s (constant) and an initial head pressure of 99.5 kPa, with a sampling frequency of 12.5 Hz. Other experimental conditions included hydrogen gas (H_2_) at 40 mL/min, makeup gas (He) at 30 mL/min, and air at 300 mL/min. Injection volume was 1 μL with a split ratio of 1:100. Data analysis was conducted using instrumental software (Clarity Chromatography, v.4.0.2). Fatty acid methyl esters (FAMEs) of nutritional significance were identified through direct comparison with retention times of reference compounds, while quantitative analysis involved calculating individual FAME percentages relative to the total chromatogram area.

#### 2.3.4. Reducing Sugars

The quantification of single and total reducing sugars contained in LF samples was determined using a High-Performance Anion-Exchange chromatography with Pulsed Amperometric Detection (HPAE-PAD) system, Thermo Scientific Dionex ICS3000 (Sunnyvale, CA, USA). The sample, diluted to 1:2000 with deionized water and filtered (nylon filter 0.20 µm), was analyzed using a chromatography system equipped with a quaternary gradient inert pump, a pulsed amperometric detector, and an AS40 automated sampler. The separation was carried out on a Dionex CarboPac PA10 analytical column (250 × 4 mm i.d.) and a CarboPac PA10 guard column (50 × 4 mm i.d.). The acquisition of chromatograms was performed using the Chromeleon Chromatography Management System, version 7.3. The equipment were sourced by Thermo Fisher Scientific (Dionex), Sunnyvale, USA All experiments were carried out at 30 °C under isocratic elution using NaOH 100 mM with a flow rate of 0.8 mL min^−1^. Analyses were performed in triplicate; analyte quantifications were conducted using external standards (calibration curve range 0.5–10.0 mg L^−1^ for glucose and fructose, 0.2–2.0 mg L^−1^ for arabinose, xylose, and rhamnose; R^2^ 0.9978), and results are reported in g L^−1^. The percentage of relative standard deviations of the peak retention times were <0.8% [18].

### 2.4. Cultivation of Phaffia rhodozyma

Freeze-dried yeasts ATCC 24202 (*Xanthophyllomyces dendrorhous*), deposited as *Phaffia rhodozyma*, were purchased from the American Type Culture Collection (Manassas, VA, USA) [19]. Microorganisms were reactivated in yeast medium (YM) agar before being pre-inoculated and maintained in YM broth. YM medium had the following composition: 3 g L^−1^ yeast extract, 3 g L^−1^ malt extract, 5 g L^−1^ peptone, 10 g L^−1^ dextrose, and 20 g L^−1^ agar. The yeast was reactivated in Petri dishes containing YM solid medium at 20 °C for 48 h. Subsequently, pre-cultures were produced in 250 mL Erlenmeyer flasks using 50 mL of YM liquid medium at 20 °C for 96 h and constant stirring of 150 rpm [20]. During the initial 24 h, there was minimal growth as the yeast adapted to the cultivation conditions and medium. From approximately 24 to 50 h, there was a rapid increase in the cell population, indicating optimal conditions for yeast proliferation. After 50 h, the growth curve reaches the plateau, signaling the transition to the stationary phase. During this period, the cell population stabilized as nutrient depletion and waste accumulation limited further growth. At the end of 96 h, the optical density (OD_600_) of the culture reached a value of 1, corresponding to the maximum cell density of 10^9^ under these conditions. The growth trend of the ATCC 24202 strain is shown in the graph, reported in Figure S1 in Supplementary Materials.

### 2.5. Direct Fermentation

#### 2.5.1. Upstream Processing

The direct fermentation was carried out in a 5 L lab scale bioreactor BIOSTAT^®^ B (Sartorius, Florence, Italy) (5 L). Several parameters were controlled to optimize the growth of the yeast and the conversion of leftover food into microbial biomass and metabolites. LF1 and LF2 were separately subjected to the fermentation process under the same conditions after being sterilized in an autoclave (FVA/A1, Fedegari, Italy) at a temperature of 121 °C for 15 min to eliminate competing microorganisms. A total of 200 g for each sample was introduced into the bioreactor. Additionally 3.5 L of sterile water was added to dilute the food slutty and achieve the desired consistency for fermentation. A small amount (0.01%) of citric acid as a descaling agent was also included to prevent any deposits during the fermentation. A concentration of 8% *v*/*v* of the strain was inoculated into the bioreactor. The inoculum size was approximately 400 mL of the total working volume. During the fermentation, pH and temperature were maintained at 5 and 21 °C, respectively. The airflow rate was 3 L·min^−1^, and the rotor speed was set at 210 rpm. The dissolved oxygen level was kept at 30% to avoid oxygen limitation. An aliquot of 0.5 L of carrot extract (EXTZ-199) was added as a fermentation promoter by providing additional nutrients or growth factors. N-hexadecane 9% *v*/*v* was included as an additional promoter for microbial activity and product yield. The fermentation process lasted for a total of 168 h (one week). In order to monitor cell growth during the process, five samplings were carried out by using optical density at 600 nm.

#### 2.5.2. Downstream Processing

The output of the fermentation process was composed of biomass and fermentation broth, which were separated through centrifugation. For both fermented samples LF1 and LF2, a biomass yield (Yx/s) of ~0.5 g biomass/g substrate with a total of around 90 g of biomass was obtained. A substrate consumption of ~90% and ~4470 g of fermentation broth were registered. In order to separate the biomass (pellet) from the supernatant, the fermentation output was centrifuged (centrifuge MF 20-R, AWEL Industries, Chateau Gontier, France) at 4 °C, 6 g for 20 min, to prevent astaxanthin degradation. The pellet was rinsed with distilled water and then centrifuged under the same previous conditions to collect the cleaned pellet. The biomass was firstly stored in darkness at 4 °C in order to maintain pigment stability. Subsequently, the dried biomass was ground into a fine powder using a mortar. The frozen biomass was suspended in 3–5 mL of dimethyl sulfoxide (DMSO), and the suspension was incubated at room temperature (21 °C) for 5 h. The residual pellet was suspended in 10 mL of acetone and incubated at 21 °C, ensuring optimal conditions for solvent-based extraction. The DMSO and acetone suspensions were combined for subsequent purification and liquid–liquid separation in an extraction funnel. Petroleum ether was added to separate the astaxanthin-containing organic layer from the aqueous phase, while sodium sulfate was added to remove water traces, ensuring a clean extraction phase. The extraction was performed at 40 °C to improve phase separation efficiency. The extracted phase, containing astaxanthin, was filtered using a 0.45 μm membrane to remove residual particles. The filtered extract was concentrated and frozen at −20 °C for storage [21]. For each sample, different aliquots of 10 μL each were taken from the crude extract for HPLC analysis. These aliquots were stored in darkness and at a low temperature to prevent degradation.

### 2.6. Quantification of Astaxanthin Using HPLC

The astaxanthin content was quantified by means of High-Performance Liquid Chromatography (HPLC) analysis that was performed using a Waters 2695 system equipped with a photodiode array (PDA) detector (Waters Corporation, Milford, MA, USA) scanning in the 200–600 nm range and set at 480 nm. The chromatographic separation was carried out on an HPLC Discovery C18 column (25 cm × 4.6 mm, 5 μm particle size, Sigma–Aldrich) under isocratic conditions at 30 °C. The mobile phase consisted of a mixture of methanol/water/dichloromethane/acetonitrile (70:4:13:13, *v*/*v*/*v*/*v*), which was filtered through a 0.45-μm membrane and degassed before use. The flow rate was maintained at 1.0 mL·min^−1^ to ensure optimal resolution and peak efficiency. The sample injection volume and the total run time were set at 10 μL and 20 min, respectively. Empower 3 software (Waters Corporation, Milford, MA, USA) was used for data acquisition and handling. A calibration curve was constructed using HPLC-grade astaxanthin standard solutions, prepared at five different concentrations (1, 5, 10, 25, and 50 mg·mL^−1^) and using methanol as the solvent. Each concentration was injected in triplicate under the same chromatographic conditions. The calibration curve exhibited a high correlation coefficient (R^2^ > 0.999) [22].

### 2.7. DPPH Assay

The antioxidant activity of astaxanthin was evaluated using the 1,1-diphenyl-2-picrylhydrazyl (DPPH) radical scavenging assay. A 0.2 mM DPPH solution was prepared by dissolving 7.89 mg of DPPH in 100 mL of methanol. Aliquots of 0.3 mL of the astaxanthin solutions at different concentrations were mixed with 2.7 mL of the DPPH solution. DPPH-scavenging activity was observed within the 5–130 mg/L range. The antioxidant capacity was quantified as mg Trolox equivalent per mL, using a Trolox standard curve (50–200 µM) prepared in 80% methanol–water solution. Spectrophotometric measurements were performed following a 30-min incubation in darkness.

The DPPH radical scavenging activity (%) was determined according to the following equation:

DPPH radical scavenging % = [(A_0_ − A_1_)/A_0_] × 100 (1)

where A_0_ represents the absorbance of the DPPH solution without the sample, and A_1_ is the absorbance measured after 30 min of incubation in the presence of astaxanthin [23].

### 2.8. Statistical Analysis

The dataset has been subjected to one-way ANOVA followed by Tukey’s honestly significant difference (HSD) test. In particular, significant differences (*p* < 0.05) within means were analyzed. The dataset is expressed as the mean ± standard deviation of triplicate measurements. XLStat statistical software (Excel 2016, ver 16.0) for Microsoft Excel was used (Microsoft Corporation, Redmond, WA, USA).

## 3. Results

### 3.1. Characterization of Raw Materials

#### 3.1.1. Physicochemical Properties

The physicochemical properties of the two analyzed samples (LF1) and (LF2) are presented in Table 1. The results are expressed as the mean ± standard deviation of the values obtained for each aliquot of LF1 and LF2, respectively. The physicochemical analysis of LF1 and LF2 revealed no statistically significant differences (*p* > 0.05) between the two samples, indicating similar overall properties. LF1 showed marginally higher values for dry residue (3.91% vs. 2.97%), lipids (10.1% vs. 8.4%), protein content (11.5% vs. 10.8%), and dietary fiber (7.52% vs. 5.43%), suggesting slightly better nutritional density. LF2, on the other hand, exhibited higher moisture content (7.03% vs. 6.91%) and lower pH (5.57 vs. 6.44), making it more acidic and hydrated. Both samples had similar ash content, reflecting comparable mineral levels. LF1’s composition makes it suitable for nutrient-dense and shelf-stable products, while LF2’s higher moisture and acidity might favor applications where hydration and fermentation are required. Overall, both samples exhibit consistent and nutritionally valuable profiles. Statistical analysis (Tukey’s HSD) shows no significant differences (*p* > 0.05) between the two samples.

#### 3.1.2. Determination of the Fatty Acid Profile

The fatty acid (FA) composition of the two analyzed samples (LF1) and (LF2) at different fermentation steps are presented in Table 2, where the content of different FAs, categorized as saturated (SFA), monounsaturated (MUFA), and polyunsaturated (PUFA), has been reported. From both LF1 and LF2, a number of five aliquots (N = 5) were analyzed in triplicate. The results are expressed as the mean ± standard deviation of the percentage values obtained for each aliquot of LF1 and LF2, respectively.

During the fermentation process, the analysis revealed distinct behaviors among fatty acid categories, with different dynamics observed for saturated fatty acids (SFAs), monounsaturated fatty acids (MUFAs), and polyunsaturated fatty acids (PUFAs). The content of saturated fatty acids (SFAs) in the two analyzed samples, LF1 and LF2, remained stable over time, with values ranging between 20.2% and 20.5%. This stability suggests that the fermentation process does not exert a significant impact on SFA composition. It can be speculated that the steady content of SFAs is due to their low reactivity, mainly attributable to the absence of double bonds in their aliphatic chains. This leads to a resistance to metabolic modifications and limited involvement in bioconversion reactions by fermentative microbes. Conversely, MUFAs showed a significant increase during the fermentation process. In sample LF1, the MUFA percentage rose from an initial 33.7% (0 h) to 43.7% at the end of the process (168 h). A similar trend was observed in LF2, where MUFAs increased from 33.6% to 43.6%. The most pronounced increments occurred between 48 and 96 h (+2.8% in LF1) and between 150 and 168 h (+1.4% in LF1), highlighting a progressive accumulation dynamic. The increase in MUFAs can be attributed to a metabolic conversion of PUFAs, facilitated by enzymatic activity from fermentative microbes. Additionally, it is likely that PUFAs are preferentially utilized as energy substrates, leaving MUFAs as accumulated products [24].

In contrast, PUFAs experienced a significant decrease during the fermentation process. In LF1, PUFA content dropped from 45.7% to 36.7%, while in LF2, the decline was from 45.7% to 36.6%. The most marked decrease occurred between 96 and 150 h (−2.9% in LF1). This trend, also supported by statistical analysis (with distinct superscripts), reflects the greater susceptibility of PUFAs to oxidation reactions and metabolic conversions. Fermentative microbes likely use PUFAs for energy metabolism or the production of useful metabolic intermediates during fermentation. Statistical analysis confirmed these observations [25]. Statistically significant differences in MUFA and PUFA percentages at different time points support the hypothesis of dynamic changes in these fatty acids during fermentation.

### 3.2. Trends of Single and Total Reducing Sugars During Fermentation

The trend of reducing sugar consumption throughout the entire fermentation process was monitored, as it was considered a crucial parameter in evaluating process efficiency. The levels of individual and total reducing sugars were quantified through HPLC analysis with amperometric detection of samples taken from the fermentation process at the following specific time intervals: 0, 24, 48, 96, 150, and 168 h. The results obtained for each sample, LF1 and LF2, are expressed as the mean and standard deviation of the values recorded for five aliquots of each sample, collected at the specified times and analyzed in triplicate. The changes in total sugar content for LF1 and LF2 samples during the fermentation process are shown in Figure S2 in Supplementary Materials. The graph highlights a steady decline in sugar levels for both samples, with LF1 demonstrating slightly faster sugar degradation compared to LF2. At the beginning of the fermentation (0 h), LF1 had a slightly higher initial sugar content (489.81 ± 11.5 g·L^−1^) compared to LF2 (448.32 ± 19.7 g·L^−1^). During the first 96 h, a significant reduction in sugar content was observed in both cases, indicating active metabolic activity, likely driven by the yeast or microorganisms involved. This phase appears to be the most critical for sugar consumption, as it shows the steepest decline. By the end of the fermentation (168 h), a substantial amount of total reducing sugars had been consumed, leaving a residual sugar content of around 137.58 g. This suggests that while fermentation was largely effective, some limitations in microbial efficiency or fermentation conditions may have prevented complete sugar utilization [26].

**Figure 1:**
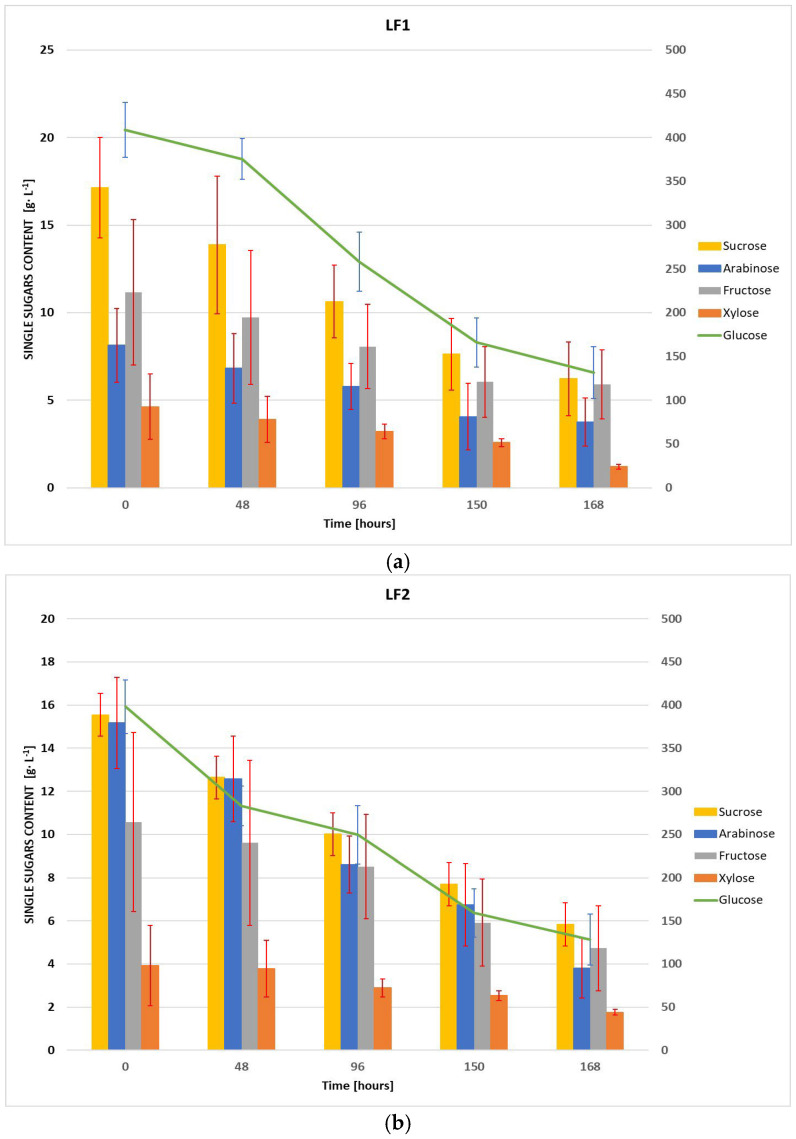
Changes of single reducing sugars content during fermentation: (**a**) LF1 sample; (**b**) LF2 sample.

In Figure 1a,b, the trends of the single sugar contents during the fermentation processes are illustrated for LF1 and LF2 samples, respectively. Each graph provides the levels of arabinose, xylose, fructose, and sucrose represented as bar plots, while the glucose trend is tracked using a green line. The concentrations of all detected sugars at different times during the fermentation are provided in Table S2 in Supplementary Materials. For both LF1 and LF2, the results are expressed as the mean ± standard deviation of the values recorded for five aliquots of each sample, collected at the specific time intervals and analyzed in triplicate. Glucose may be considered the primary energy source for the microorganism, with 67.8% of consumption by the end of fermentation for both samples. In fact, the glucose levels at the start of fermentation process were significantly high (408.86 ± 31.17 g·L^−1^ vs. 398.25 ± 30.86 g·L^−1^, in LF1 and LF2 respectively) with a steep decline over time, reaching about 250 g·L^−1^ at 96 h and stabilizing at 130 g·L^−1^ ca. at 168 h. The other single sugars followed similar patterns. Sucrose showed a similar depletion pattern, with a notable 63% and 62% reduction in LF1 and LF2, respectively. Regarding arabinose, the data suggests a distinct difference in its metabolism between LF1 and LF2. While initial arabinose concentrations were higher in LF2 compared to LF1, both samples reached similar final concentrations, with LF2 exhibiting a higher overall consumption rate (75% vs. 54%). Fructose showed lower consumption compared to glucose and sucrose, with 47% in LF1 and 55% in LF2, likely due to its delayed metabolism after glucose depletion. Xylose, present in minimal initial amounts, exhibited a variable consumption pattern between the two samples, with a 74% reduction in LF1 and 55% in LF2.

### 3.3. Astaxanthin Production

**Figure 2:**
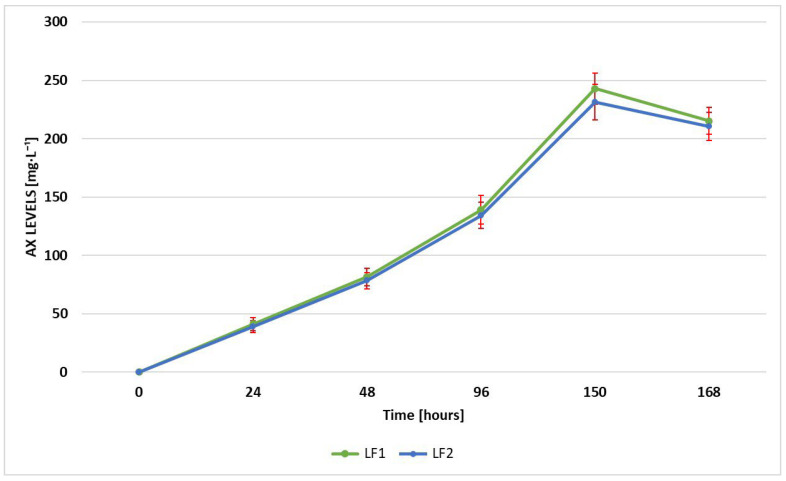
AX production during fermentation [mg·L^−1^] (LF1 vs. LF2).

**Figure 3:**
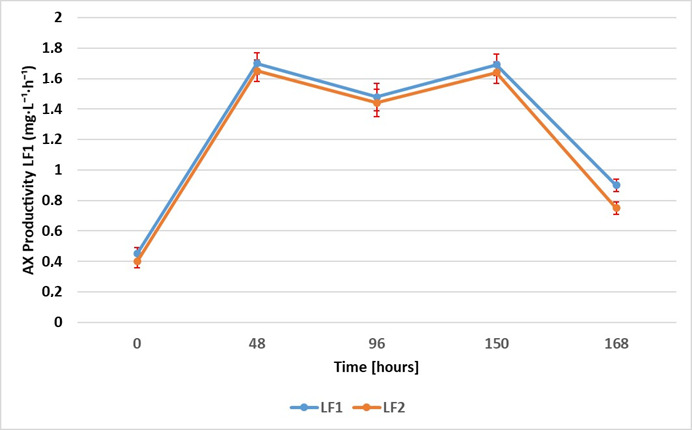
AX productivity during fermentation [mg·L^−1^·h^−1^] (LF1 vs. LF2).

Astaxanthin production was monitored during the entire fermentation process. The levels of AX were quantified through HPLC analysis at various time intervals, namely, 0, 24, 48, 96, 150, and 168 h. The results obtained for each sample, LF1 and LF2, were expressed as the mean ± standard deviation of the values recorded for five aliquots of each sample, collected at the specified times and analyzed in triplicate. In order to evaluate the efficacy of the fermentation setup, the AX content and productivity were evaluated (see Figure 2 and Figure 3). In particular, the productivity consists of the amount of astaxanthin (mg) generated per unit of time (hours) and the volume of fermentation output (liters). The graph in Figure 2 shows that both samples had a similar trend of increasing in AX concentration over time. The production rate appears to accelerate initially, reaching a peak at around 150 h, followed by a slight decline by 168 h. The maxima in AX concentration were 242.96 ± 15.41 mg·L^−1^ for LF1 and 231.26 ± 13.27 mg·L^−1^ for LF2, at around 150 h, identified as the most efficient time of the whole fermentation process developed. After 150 h, both samples had a slight decrease in astaxanthin levels, which could indicate the onset of nutrient depletion or changes in environmental conditions affecting production [26]. As shown in Figure 3, LF1 and LF2 exhibited a comparable productivity trend, which is characterized by an initial increase until 48 h, followed by a slight decrease at 96 h; then, productivity increased again, attaining its maximum at 150 h (1.69 ± 0.04 mg·L^−1^·h^−1^ for LF1 and 1.64 ± 0.03 mg·L^−1^·h^−1^ for LF2). The sharp decline after 150 h suggests potential factors such as substrate depletion, an accumulation of inhibitory by-products, or alterations in microbial or metabolic activity, leading to reduced production efficiency.

### 3.4. Antioxidant Activity of the Fermentation Products

The antioxidant activity of the fermented products was investigated using a 2,2-diphenyl-1picrylhydrazyl (DPPH) assay, a method based on a reduction in the stable DPPH radical [27]. The scavenging efficiency of antioxidants was determined using the spectrophotometric measurements of reduction in absorbance at 517 nm. In order to investigate the impact of the fermentation process on antioxidant properties, LF1 and LF2 were collected after 150 h from the start of the fermentation process and analyzed. The findings revealed a significant increase in DPPH-scavenging activity within the 5–130 mg/L range. Antioxidant capacity was quantified as mg Trolox equivalent per mL, utilizing a Trolox standard curve (50–200 µM) prepared in an 80% methanol–water solution. The results indicated that LF1 exhibited a radical scavenging activity of 66.85 ± 0.26%, while LF2 showed significantly higher activity at 78.42 ± 0.69%, corresponding to 0.18 ± 0.01 mgEq/mL and 0.22 ± 0.02 mgEq/mL of Trolox, respectively. A direct correlation can be drawn between the fermentation process and enhanced antioxidant activity. Additionally, both fermented products exhibited a 26% higher antioxidant concentration compared to standard synthetic astaxanthin, underscoring the potential of fermentation as a recommended approach for enhancing the production of antioxidant compounds.

## 4. Discussion

The implementation of a physical and thermomechanical pretreatment protocol for raw materials represents an alternative and more sustainable approach compared to acid and enzymatic hydrolysis, which are commonly employed for the pre-digestion of organic waste. The latter methods present significant economic and environmental criticisms, particularly due to the use of chemicals, high costs, and difficulties in enzyme stabilization. The pretreatment protocol was selected to ensure a low environmental impact for the process. In fact, drying, grinding, and sonication require only electrical energy and do not necessitate chemical reagents, thereby reducing the risk of secondary pollution and lowering operational costs [28]. Specifically, the sonication operating conditions were designed to enhance nutrient availability by breaking down cellular structures and facilitating the release of organic compounds, thereby making nutrients more accessible to the microorganism involved in fermentation [29]. Furthermore, physical and thermomechanical pretreatment prevents the formation of inhibitory compounds, such as furfural and hydroxymethylfurfural, which are typical byproducts of acid hydrolysis [30]. A sustainable pretreatment is certainly a preferable solution in the prospect of industrial-scale process transfer. However, this preliminary study requires further investigation, particularly regarding the optimization of operating conditions and the reduction in process energy consumption. The similar chemico-physical characteristics of LF1 and LF2 samples are related to their comparable composition. However, LF1 showed a slightly higher nutritional density, while LF2 was more acidic and hydrated. The FAs profile, assessed during the fermentation, highlights that MUFAs increased significantly, whereas PUFAs decreased, reflecting the metabolic conversions. The increase in MUFAs could be attributed to the enzymatic desaturation and elongation of SFAs, as well as the microbial activity favoring their accumulation. On the other hand, the reduction in PUFAs suggests oxidative degradation or β-oxidation processes, which are commonly observed in fermentation due to microbial metabolism and the susceptibility of PUFAs to oxidation [24,25]. Sugar consumption was rapid in the early stages of fermentation, and LF1 demonstrated a slightly faster degradation. AX production and productivity reached the maximum point at 150 h for both samples, followed by a decline, indicating optimal efficiency at that time. Overall, both samples exhibited promising profiles for nutritional and fermentative applications. The data suggest that the LF1 and LF2 samples primarily utilize glucose, fructose, and sucrose as carbon sources, while the protein content (11.5% LF1 vs. 10.8% LF2) indicated the presence of a useful nitrogen source.

The C/N ratio can be approximately estimated at around 20 for both samples by considering carbohydrates as the main contributors to carbon and the protein content as the main source of nitrogen. The C/N ratio significantly impacted fermentation, influencing both astaxanthin content and the overall productivity of the process due to its balanced microbial growth with the synthesis of secondary metabolites, such as astaxanthin. From the comparison with other studies, it emerges that various fermentation approaches have been implemented to obtain astaxanthin from different natural matrices. The criteria used for comparison were as follows: (i) availability, cost-effectiveness, and homogeneity of the raw materials used as substrates; (ii) type of fermentation approach employed; (iii) economical and environmental sustainability of the processes (iv) efficiency of the processes. A study by Jiang et al. (2017) identified Jerusalem artichoke as a culture medium for *Phaffia rhodozyma* Y119 and implemented both batch and fed-batch fermentation processes [10]. However, the astaxanthin contents obtained using this homogeneous but not easily accessible matrix were lower in both processes compared to our study, where the production exceeded 230 mg·L^−1^. In the work of Jiang et al., the total carotenoid content reached the peak at 107.78 mg·L^−1^ after 108 h in the batch process and at 179.50 mg·L^−1^ after 152 h in the fed-batch process. It is important to highlight that the initial concentration of reducing sugars in the Jerusalem artichoke medium was approximately 30 g·L^−1^, which is significantly lower than the concentrations found in our study [10]. This difference underscores the advantages of our matrix, which is not only heterogeneous, readily available, and cost-free but also highly suitable to direct fermentation processes, providing a more efficient substrate for astaxanthin and carotenoid production.

Another study focused on the production of astaxanthin through the cultivation of *Phaffia rhodozyma* ATCC 74219 on a substrate made of sweet sorghum juice and bagasse. The strength of this work lies in the use of a homogeneous, readily available, and cost-effective byproduct as the raw material. However, the process achieved a maximum astaxanthin content and productivity of 48.89 mg·L^−1^ and 0.291 mg·L^−1^·h^−1^ at 192 h from the start of the process (production peak), which are significantly lower than those in our study. Furthermore, the entire approach described by Stoklosa et al. is less environmentally and economically sustainable. Before fermentation in the bioreactor, the sweet sorghum undergoes chemical pretreatment with anhydrous ammonia, followed by enzymatic hydrolysis with cellulase and hemicellulose [26]. Finally, the results of this study reveal a significant antioxidant activity in the fermentation products, as demonstrated by the DPPH scavenging activity values exceeding 65% in both samples. The identified fermentation conditions have, therefore, positively influenced the antioxidant properties of the output of processes. Moreover, these findings are consistent with previous studies that utilized homogeneous food waste from corn, wheat, and mesquite pods as substrates for fermentation [31,32,33].

## 5. Conclusions

This study contributed to demonstrating the potential of leftover food as an innovative substrate for scalable and efficient astaxanthin production, contributing to both economic feasibility and environmental sustainability. The circular valorization of heterogeneous and cost-free raw materials, the use of low-impact pretreatment techniques based on physical and mechanical processes, and the development of a direct fermentation approach have been key factors in the achievement of the predefined aims of this work with relevant levels of efficiency and effectiveness in the process. Although this is a preliminary study and the experimental plan was conducted on a lab scale, the results suggest that it can provide an interesting starting point for further future developments.

## Figures and Tables

**Table 1:** Physicochemical properties of pretreated raw materials.

Sample	Physicochemical Properties						
	pH	Moisture [%]	Dry Residue	Ashes	Total Lipids [%]	Protein Content	Dietary Fiber
LF1 (*n* = 5) *mean ± st. dev*	6.44 ± 0.4 ^a^	6.91 ± 0.7 ^a^	3.91 ± 0.04 ^a^	1.41 ± 0.09 ^a^	10.1 ± 1.1 ^a^	11.5 ± 0.9 ^a^	7.52 ± 0.6 ^a^
LF2 (*n* = 5) *mean ± st. dev*	5.57 ± 0.8 ^a^	7.03 ± 0.9 ^a^	2.97 ± 0.09 ^a^	1.22 ± 0.05 ^a^	8.4 ± 0.6 ^a^	10.8 ± 0.7 ^a^	5.43 ± 0.6 ^a^

Different superscript letters indicate significantly different values (*p* < 0.05 using post hoc Tukey’s HSD test); the same superscript letters in the same column indicate not significantly different values (*p* > 0.05 using post hoc Tukey’s HSD test).

**Table 2:** Fatty acid profiles of LF1 and LF2 categorized as saturated (SFA), monounsaturated (MUFA), and polyunsaturated (PUFA).

Fermentation Steps [Hours]	SFA [%]		MUFA [%]		PUFA [%]	
	LF1 (N = 5) *Mean ± St. Dev*	LF2 (N = 5) *Mean ± St. Dev*	LF1 (N = 5) *Mean ± St. Dev*	LF2 (N = 5) *Mean ± St. Dev*	LF1 (N = 5) *Mean ± St. Dev*	LF2 (N = 5) *Mean ± St. Dev*
0	20.5 ± 0.91 ^a^	20.4 ± 1.03 ^a^	33.7 ± 1.61 ^a^	33.6 ± 1.81 ^a^	45.7 ± 0.09 ^a^	45.7 ± 2.22 ^a^
48	20.4 ± 0.96 ^a^	20.4 ± 1.03 ^a^	36.6 ± 1.72 ^b^	36.4 ± 1.69 ^b^	43.1 ± 1.13 ^b^	43.0 ± 2.22 ^b^
96	20.5 ± 0.46 ^a^	20.4 ± 1.44 ^a^	39.4 ± 1.62 ^c^	39.3 ± 1.83 ^c^	40.6 ± 1.09 ^c^	40.5 ± 2.22 ^c^
150	20.5 ± 1.61 ^a^	20.4 ± 2.01 ^a^	42.3 ± 1.65 ^d^	42.5 ± 1.86 ^d^	37.7 ± 0.09 ^d^	37.6 ± 2.25 ^d^
168	20.5 ± 1.12 ^a^	20.2 ± 1.18 ^a^	43.7 ± 1.16 ^d^	43.6 ± 1.94 ^d^	36.7 ± 1.21 ^d^	36.6 ± 2.13 ^d^

Different superscript letters indicate significantly different values (*p* < 0.05 using post hoc Tukey’s HSD test); the same superscript letters in the same column indicate not significantly different values (*p* > 0.05 using post hoc Tukey’s HSD test).

## Data Availability

The original contributions presented in this study are included in the article/Supplementary Material. Further inquiries can be directed to the corresponding author.

## Supplementary Materials

The following supporting information can be downloaded at: https://www.mdpi.com/article/10.3390/foods14071232/s1, Table S1: Rough composition of the collected samples LF1 and LF2; Table S2: Single reducing sugars content during fermentation in LF1 and LF2 samples. Figure S1: Growth trend of the ATCC 24202 strain during the range of cultivation 0–96 h at 20 °C; Figure S2: Changes of total reducing sugars (TRS) content during fermentation (LF1 vs. LF2).
