# Supplementary material for: Leftover Food as a Sustainable Source of Astaxanthin Through Fermentation Using Phaffia rhodozyma
Source: Foods. 2025 Mar 31;14(7):1232. doi: 10.3390/foods14071232 (PMC11988289; doi:10.3390/foods14071232)
Supplement: Supplementary file 1 [file foods-14-01232-s001.zip › foods-3489771-supplementary.pdf]

Table S1- Composition of the collected samples LF1 and LF2

Composition	LF1 [weight percentage]	LF2 [weight percentage]
Rice, maize and pasta parboiled	45	43
Used oils	5	7
Raw and cooked vegetables (lettuce leaves, potatoes and spinach, beets, peas and carrots)	32	35
Fruit peels (bananas, apples and pear)	18	15

Figure S1- Growth trend of the ATCC 24202 strain during the range of cultivation 0-96h at 20 °C.

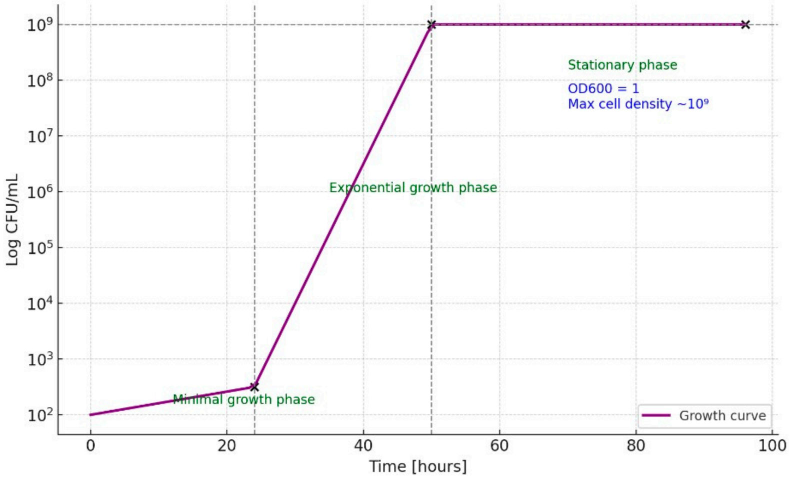

Figure S2. Changes of total reducing sugars (TRS) content during fermentation (LF1 vs LF2)

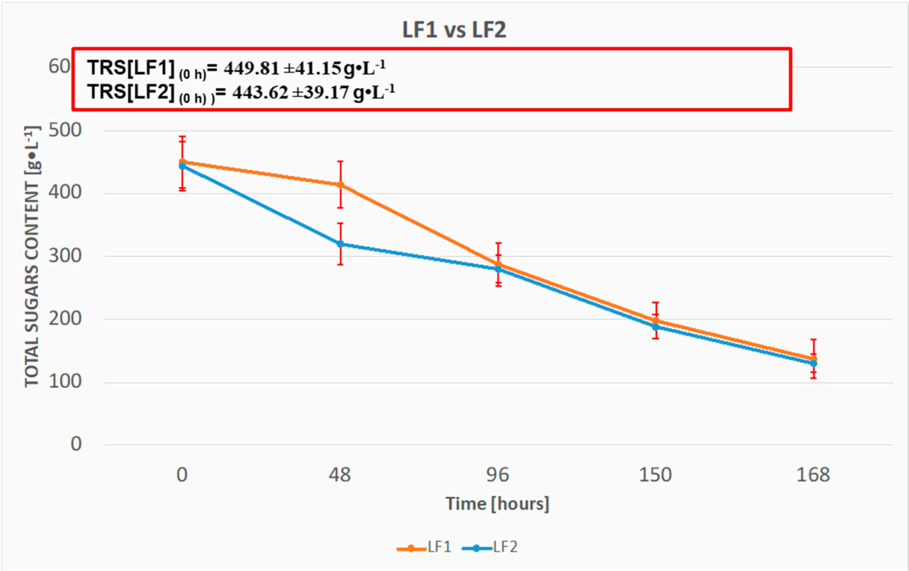

Table S2- Single reducing sugars content during fermentation in LF1 and LF2 samples.

Fermentation steps [hours]	Glucose [g· L^{-1}]		Sucrose [g· L^{-1}]		Arabinose [g· L^{-1}]		Fructose [g· L^{-1}]		Xylose [g· L^{-1}]	
	LF1 (N=5) *mean ± st. dev*	LF2 (N=5) *mean ± st. dev*	LF1 (N=5) *mean ± st. dev*	LF2 (N=5) *mean ± st. dev*	LF1 (N=5) *mean ± st. dev*	LF2 (N=5) *mean ± st. dev*	LF1 (N=5) *mean ± st. dev*	LF2 (N=5) *mean ± st. dev*	LF1 (N=5) *mean ± st. dev*	LF2 (N=5) *mean ± st. dev*
0	408.86±31.17	398.25±30.86	17.13±2.87	15.55±2.79	8.14±2.11	15.17±2.15	11.17±4.15	10.58±4.13	4.63±1.87	3.93±1.94
48	375.56±23.14	283.16±29.15	13.87±3.92	12.64±3.09	6.82±1.98	12.58±2.89	9.73±3.83	9.61±3.64	3.91±1.31	3.78±1.22
96	258.43±33.67	249.41±24.15	10.64±2.07	10.01±2.36	5.79±1.32	8.61±1.75	8.07±2.41	8.52±2.89	3.21±0.42	2.89±0.53
150	166.14±28.16	159.24±24.44	7.63±2.04	7.71±2.04	4.07±1.91	6.74±2.01	6.05±2.01	5.92±2.11	2.58±0.22	2.53±0.32
168	131.62±29.57	128.19±27.51	6.23±2.11	5.83±2.56	3.75±1.38	3.81±1.42	5.91±1.98	4.73±1.71	1.21±0.13	1.76±0.17
